# Induction of Apoptosis and Autophagy in Breast Cancer Cells by a Novel HDAC8 Inhibitor

**DOI:** 10.3390/biom9120824

**Published:** 2019-12-04

**Authors:** Chang-Fang Chiu, Hsien-Kuo Chin, Wei-Jan Huang, Li-Yuan Bai, Hao-Yu Huang, Jing-Ru Weng

**Affiliations:** 1 Division of Hematology and Oncology, Department of Internal Medicine, China Medical University Hospital, Taichung 40447, Taiwan; d5686@mail.cmuh.org.tw (C.-F.C.); lybai6@gmail.com (L.-Y.B.); 2 Cancer Center, China Medical University Hospital, Taichung 40415, Taiwan; 3 College of Medicine, China Medical University, Taichung 40402, Taiwan; 4 Division of Cardiovascular Surgery, Department of Surgery, Kaohsiung Armed Forces General Hospital, Kaohsiung 80284, Taiwan; cvschin@gmail.com; 5 Department of Marine Biotechnology and Resources, National Sun Yat-sen University, Kaohsiung 80424, Taiwan; 6 Graduate Institute of Pharmacognosy, College of Pharmacy, Taipei Medical University, Taipei 11031, Taiwan; wjhuang@tmu.edu.tw; 7 Doctoral Degree Program in Marine Biotechnology, National Sun Yat-sen University, Kaohsiung 80424, Taiwan; 8 Graduate Institute of Natural Products, College of Pharmacy, Kaohsiung Medical University, Kaohsiung 80715, Taiwan

**Keywords:** histone deacetylase, HDAC8-selective inhibitor, breast cancer, apoptosis, autophagy, PPARγ, ROS

## Abstract

Epigenetic therapy has been demonstrated to be a viable strategy for breast cancer treatment. In this study, we report the anti-tumor activity of a hydroxamate-based histone deacetylase (HDAC)8-selective inhibitor, HMC, in breast cancer cells. MTT assays showed that HMC inhibited cell viability of MCF-7 and MDA-MB-231 cells with IC_50_ values of 7.7 μM and 9.5 μM, respectively. HMC induced caspase-dependent apoptosis in MCF-7 cells, which was associated with its ability to modulate a series of cell survival-related signaling effectors, including Akt, mTOR, Bax, Mcl-1, and Bcl-2. Additionally, HMC was capable of activating PPARγ, which was accompanied by reduced expression of PPARγ target gene products, such as cyclin D1 and CDK6. HMC increased the production of ROS in MCF-7 cells, which could be partially reversed by the cotreatment with a ROS scavenger (*N*-acetylcysteine or glutathione). Furthermore, HMC induced autophagy, as characterized by the formation of acidic vesicular organelles and autophagic biomarkers including LC3B-II and Atg5. Notably, pharmacological blockade of autophagy by 3-MA or CQ could attenuate HMC-induced apoptosis, suggesting that autophagy played a self-protective role in HMC-induced cell death. Together, these data suggest the translational potential of HMC to be developed into a potential therapeutic agent for breast cancer therapy.

## 1. Introduction

Increasing incidences and mortality of breast cancer still remains an unresolved issue in women’s health, with 2.1-million new cases and over 600,000 deaths worldwide in 2018 [1]. Family history of breast cancer, inherited BRCA1 and/or BRCA2 mutations, alcohol intake, and exogenous hormone intake are known risk factors underlying the elevated incidence rate of breast cancer [2]. Despite recent advances in the development of targeted therapy, the overall survival in advanced breast cancer patients remains low at approximately 18% [3], indicating an urgency in developing new therapeutic strategies.

As substantial evidence has linked dysregulation of histone deacetylases with tumorigenesis [4,5], HDAC inhibitors have emerged as potential therapeutic agents for multiple types of human cancer due to their diverse modes of antitumor mechanisms [6]. For example, the FDA-approved HDAC inhibitor suberoylanilide hydroxamic acid (SAHA, vorinostat) [7] was reported to inhibit cell growth by increasing HSP60 nitration and reactive oxygen species (ROS) production in lung cancer cells [8]. SAHA was also shown to synergize with the PARP inhibitor Olaparib in triple-negative breast cancer (TNBC) in vitro and in vivo by inducing apoptosis and autophagic cell death [9]. Evidence has shown clinical benefits of using SAHA in 40% of advanced tamoxifen-resistant breast cancer patients [10].

**Figure 1:**
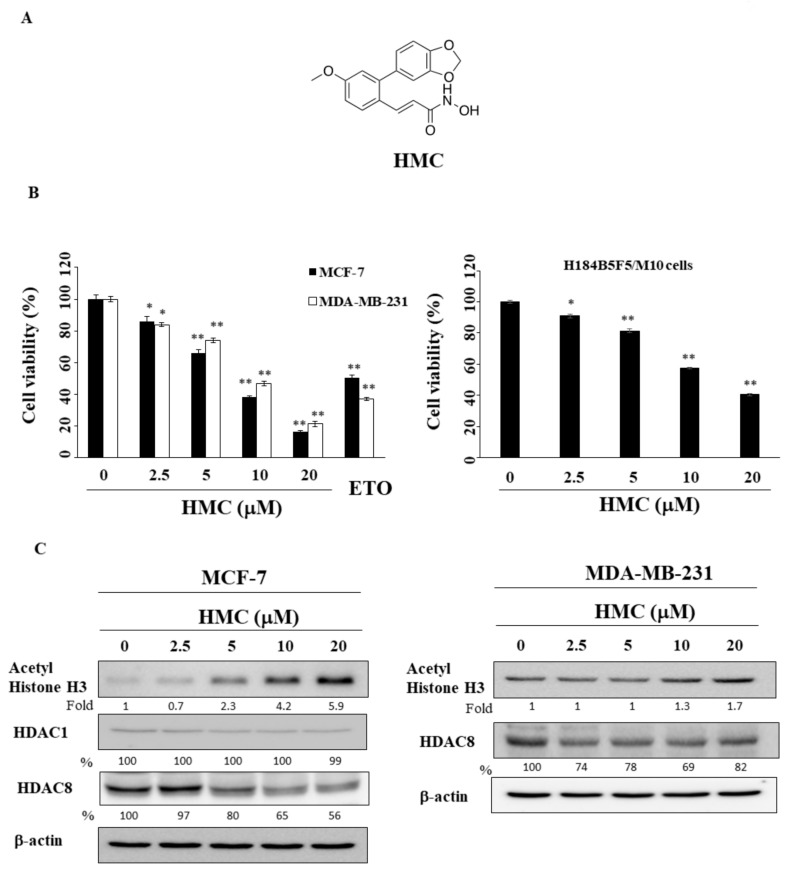
Antiproliferative effects of HMC in breast cancer cells and normal human breast epithelial cells. (**A**) The chemical structure of HMC. (**B**) Left panel, cells were treated with DMSO or HMC at the indicated concentration for 48 h, cell viability (MTT assay) were tested. Positive control: 20 μM or 30 μM etoposide was used as positive control. (MCF-7 or MDA-MB-231cells). Right panel, Non-tumorgenic human breast epithelial cell line H184B5F5/M10 was treated with HMC for 48 h, and cell viability was determined by MTT assay. Points, means; bars, SD (n = 4–6). * *p* < 0.05, ** *p* < 0.01. (**C**) Western blot analysis of acetyl Histone H3, HDAC1, and HDAC8 in HMC-treated cells for 48 h. Left panel, MCF-7 cells. Right panel, MDA-MB-231 cells. The values in percentage or fold denote the relative intensity of protein bands of HMC treated samples to that of the respective DMSO vehicle control after being normalized to the respective internal reference (β-actin).

Among 11 Zn^2+^-dependent HDAC isozymes, HDAC8 was found immunoreactive in 85% of breast cancer patients [11,12]. An et al. demonstrated that HDAC8 inhibitor PCI34051 suppressed the migration of breast cancer cells by facilitating the degradation of YAP [13]. In this study, we report the characterization of the anti-tumor activity and underlying mechanisms of a novel HDAC8 inhibitor, (*E*)-*N*-hydroxy-4-methoxy-2-(3,4-methylenedioxyphenyl)cinnamide (HMC) (Figure 1A and Figure S1) [14], in breast cancer cells.

## 2. Results

### 2.1. HMC Inhibits the Viability of Breast Cancer Cells and Modulates HDAC Expression

We used two breast cancer cell lines, MCF-7 and MDA-MB-231, to interrogate the anti-proliferative effect of HMC. MTT assays showed that the dose-dependent suppressive effect of HMC on the viability of MCF-7 and MDA-MB-231 cells with IC_50_ values of 7.7 μM and 9.5 μM, respectively, after 48 h of treatment (Figure 1B; etoposide as the positive control). Additionally, the non-tumorgenic human breast epithelial cell line H184B5F5/M10 was less sensitive to HMC with an IC50 value of 14.1 μM (right panel of Figure 1B). Western blot analysis of HMC-treated MCF-7 and MDA-MB-231 cell lysates shows that this antiproliferative effect was associated with histone H3 hyperacetylation, reflecting the effect of HDAC8 inhibition (Figure 1C). Interestingly, HMC treatment led to decreases in HDAC8 expression which is similar to the finding of PCI34051 in angiotension-II-induced hypertensive mice [15], while the level of HDAC1 remained largely unchanged in MCF-7 cells (Figure 1C).

### 2.2. HMC Induces Apoptosis

**Figure 2:**
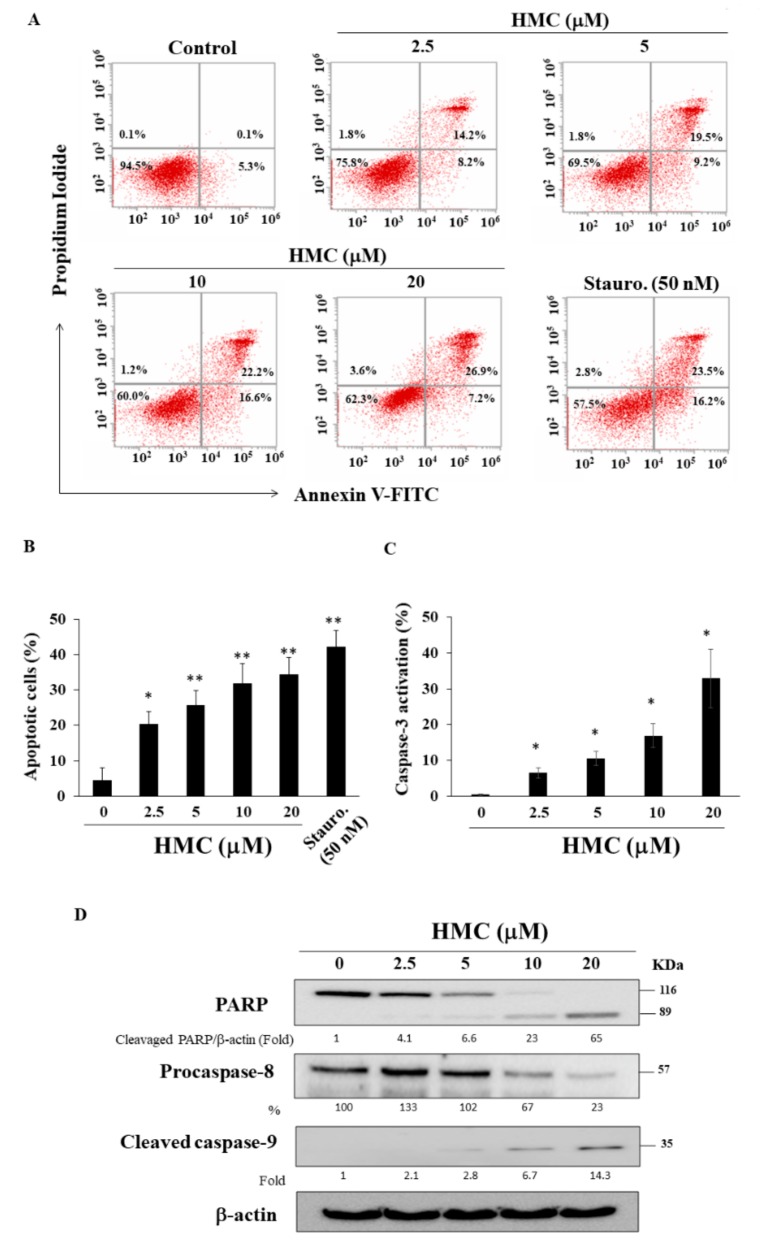
HMC induces apoptosis in MCF-7 cells. (**A**) Cells were treated with DMSO or HMC or staurosporine (Stauro.) for 48 h, and stained with propidium iodide (PI)/annexin V. (**B**) Statistically analysis of apoptotic cells (Q2+Q4) after the treatment of HMC for 48 h. Points, means; bars, SD (n = 4) * *p* < 0.05, ** *p* < 0.01. (**C**) Caspase-3 activation after the treatment of HMC for 48 h. Cells were collected after the treatment of DMSO or HMC and detected using flow cytometry as Materials and methods. Points, means; bars, SD (n = 3) * *p* < 0.05. (**D**) Expression of PARP, procaspase-8, and cleaved caspase-9 in HMC-treated cells. Total cell lysates were collected as Materials and methods. The values in percentage or fold denote the relative intensity of protein bands of HMC treated samples to that of the respective DMSO vehicle control after being normalized to β-actin.

Several lines of evidence indicate that the antiproliferative effect of HMC was attributable to its ability to induce apoptosis in MCF-7 cells. For example, flow cytometric analysis of Annexin V/PI staining shows increases in annexin V-positive cells in response to HMC treatment in a concentration-dependent manner (Figure 2A,B; staurosporine as the positive control). In addition, flow cytometry demonstrated that HMC dose-dependently increases caspase-3 activities in MCF-7 cells (Figure 2C), and Western blot analysis showed increased levels of the cleavage PARP and caspase-9, accompanied by decreased expression of procaspase-8 (Figure 2D).

### 2.3. HMC Inhibits the Akt/mTOR Signaling Pathway and Activates PPARγ

**Figure 3:**
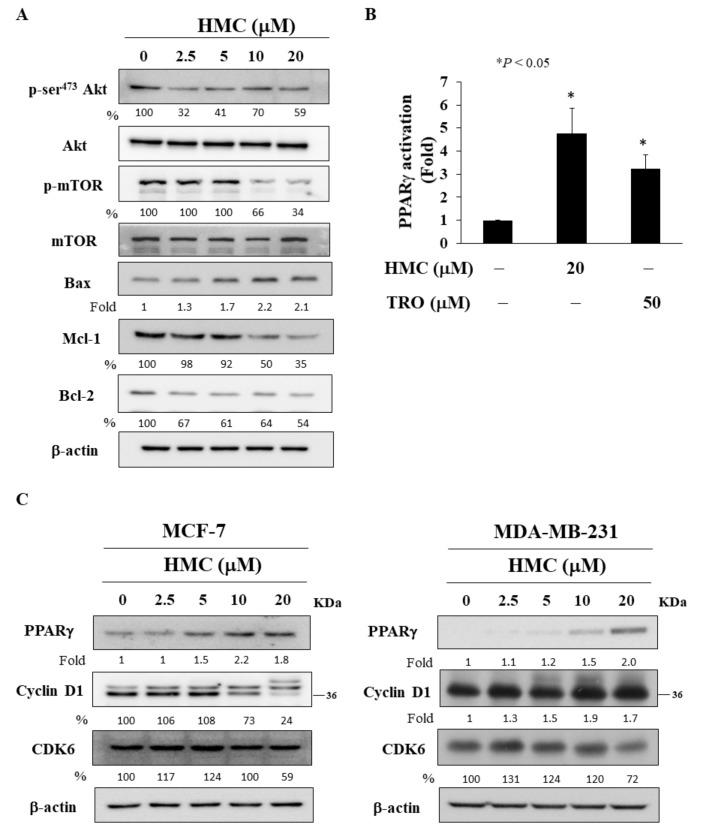
HMC modulates the expression of various biomarkers in breast cancer cells. (**A**) Phosphorylation/expression of Akt, mTOR, Bax, Mcl-1, and Bcl-2 after the treatment of HMC in MCF-7 cells. (**B**) *PPARγ* promoter transactivation in HMC-treated MCF-7 cells. 50 μM troglitazone (TRO) was used as positive control. (**C**) Levels of PPAR*γ*, cyclin D1, and CDK6 in HMC-treated cells for 48 h. Left panel, MCF-7 cells. Right panel, MDA-MB-231 cells. The values in percentage or fold denote the relative intensity of protein bands of HMC treated samples to that of the respective DMSO vehicle control after being normalized to the respective internal reference (total respective protein or β-actin).

Previously, it has been reported that the pan-HDAC inhibitor LAQ824 inhibited cell growth, in part, through the inhibition of Akt activation in prostate cancer cells [16,17]. In light of the importance of Akt in breast cancer tumorigenesis and metastasis [16,17], we analyzed the effect of HMC on the activation status of Akt signaling. Western blotting revealed that HMC treatment led to decreased phosphorylation of Akt and it’s down-stream effector mTOR in MCF-7 cells (Figure 3A). In addition, HMC up-regulated the expression of the pro-apoptotic protein Bax, accompanied by reduced expression of the anti-apoptotic proteins Mcl-1 and Bcl-2 (Figure 3A).

It has been reported that pharmacological inhibition of HDACs led to the activation of the peroxisome proliferator-activated receptor (PPAR)γ, a member of nuclear receptors associated with lipogenesis and cell metabolism [18]. In addition, the HDAC8 inhibitor NCC170 was shown to ameliorate idiopathic pulmonary fibrosis, in part, by increasing PPARγ expression [19]. Here, the effect of HMC on PPARγ was assessed using an established PPRE-luciferase reporter assay in MCF7- cells [20]. Compared with the known PPARγ agonist troglitazone, HMC showed a greater degree of PPARγ promotor transactivation in MCF-7 cells (Figure 3B). Western blot analysis showed that HMC increased PPARγ expression in MCF-7 cells, while decreasing the levels of the PPARγ-targeted gene products cyclin D1 and CDK6, both of which are associated with cell cycle regulation in MCF-7 cells [21,22] (Figure 3C). The expression of cyclin D1 and CDK6 remained unchanged in MDA-MB-231 cells treated with HMC for 48 h (Figure 3C).

### 2.4. HMC Increases ROS Generation

**Figure 4:**
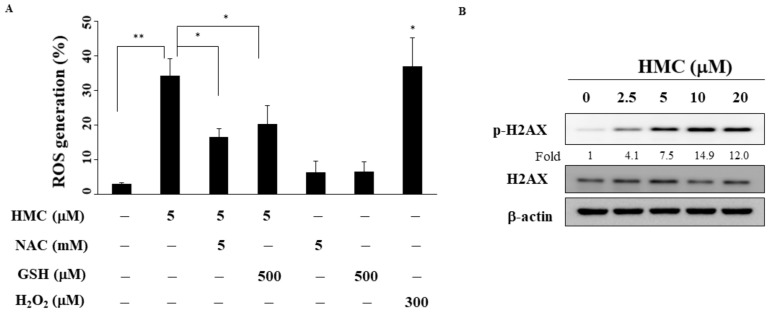
HMC increased reactive oxygen species (ROS) production. (**A**) Cells were treat with HMC alone or in combination of 5 mM *N*-acetylcysteine (NAC) or 500 μM glutathione (GSH) for 24 h. 300 μM H_2_O_2_ was used as positive control. SD (n = 3) * *p* < 0.05, ** *p* < 0.01. (**B**) Effects of HMC on the phosphorylation and expression of H2AX in MCF-7 cells. The values in fold denote the relative intensity of protein bands of HMC treated samples to that of the respective DMSO vehicle control after being normalized to the respective internal reference (total respective protein).

Previous studies have linked ROS production with the antiproliferative effect of pan-HDAC inhibitors [23,24]. As shown in Figure 4A, HMC increased ROS production in MCF-7 cells after 24 h of treatment (H_2_O_2_ as the positive control). In addition, pre-treatment with an ROS inhibitor, *N*-acetylcysteine (NAC) or glutathione (GSH), for 15 min could reverse HMC-induced ROS generation (Figure 4A). We also examined the antiproliferative effects of HMC with or without NAC or GSH in MCF-7 cells using MTT assay (S2, Figure S2). Although HMC reduced the cell viability, addition of NAC or GSH did not increase the HMC-mediated cytotoxicity. Furthermore, HMC increased the phosphorylation of H2AX, a biomarker in response to DNA damage [25], in MCF-7 cells (Figure 4B).

### 2.5. HMC Induces Autophagy

**Figure 5:**
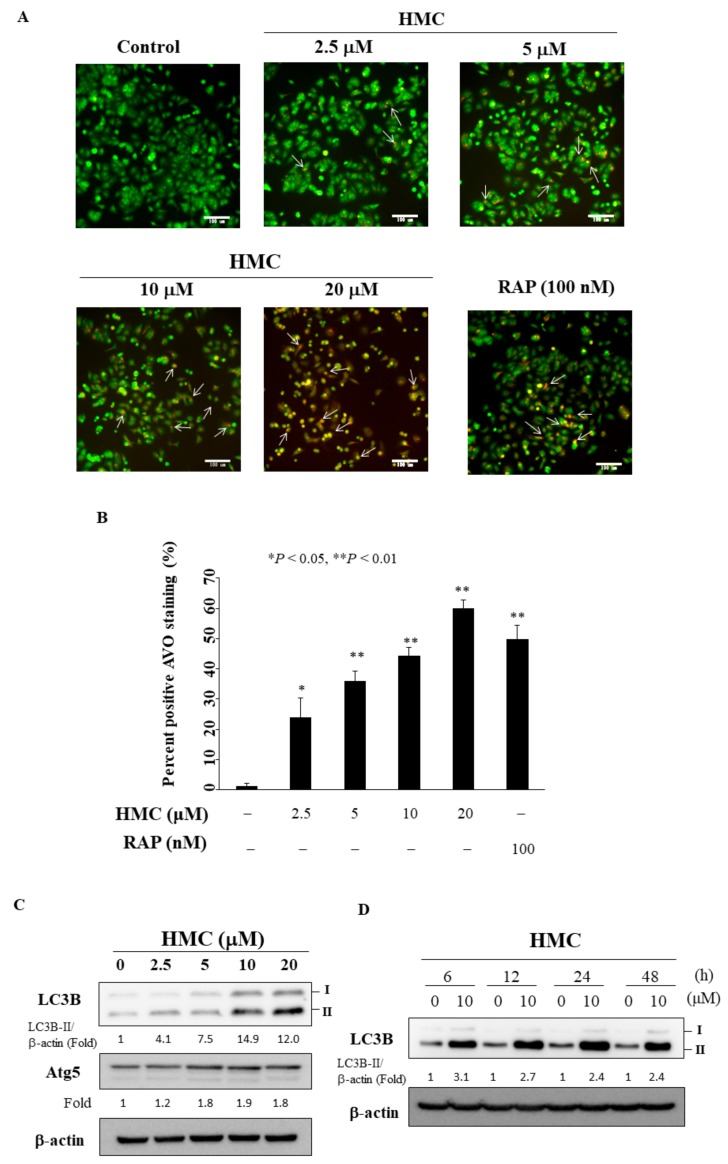
HMC induces autophagy. (**A**) Fluorescence microscopy following acridine orange staining revealed an increase in the number of cytoplasmic acidic vesicular organelles (AVOs) in MCF-7 cells for 24 h. 100 nM Rapamycin (RAP) was used as positive control. arrows: acidic vesicular organelles. magnification: 200×. (**B**) Quantitative data calculated percentage of AVO staining cells after the treatment of HMC. At least 100 cells from each treatment group were calculated per image under fluorescence microscopy. Data are represented as the mean ± SD. * *p* < 0.05, ** *p* < 0.01. (**C**) Effect of HMC on the expression of LC3B and Atg5 in MCF-7 cells. (**D**) Time-dependent effect of HMC on the expression of LC3B. The values in percentage or fold denote the relative intensity of protein bands of HMC treated samples to that of the respective DMSO vehicle control after being normalized to β-actin.

Substantial evidence has shown the ability of pan-HDAC inhibitors to promote autophagy [26,27]. During autophagy, the formation of acidic vesicular organelles (AVOs) is one of the characteristic features of cells engaged in autophagy in response to starvation or radiation [28]. Thus, we examine drug-induced cellular acidification by using acridine orange staining, in which cytoplasm fluorescence changed from bright green to bright red. As shown in Figure 5A,B, the generation of AVOs increased after the treatment of HMC in a concentration-dependent manner in MCF-7 cells (rapamycin as the positive control). In addition, immunoblotting shows HMC-induced increases in the expression of LC3B-II and autophagy-related (Atg)5 in MCF-7 cells (Figure 5C), both of which are important markers for autophagosome formation [29,30]. In addition, time-course experiments demonstrated that LC3B-II expression increased after 6 h of HMC treatment (Figure 5D).

### 2.6. Inhibition of Autophagy Reversed HMC-Induced Apoptosis in MCF-7 Cells

**Figure 6:**
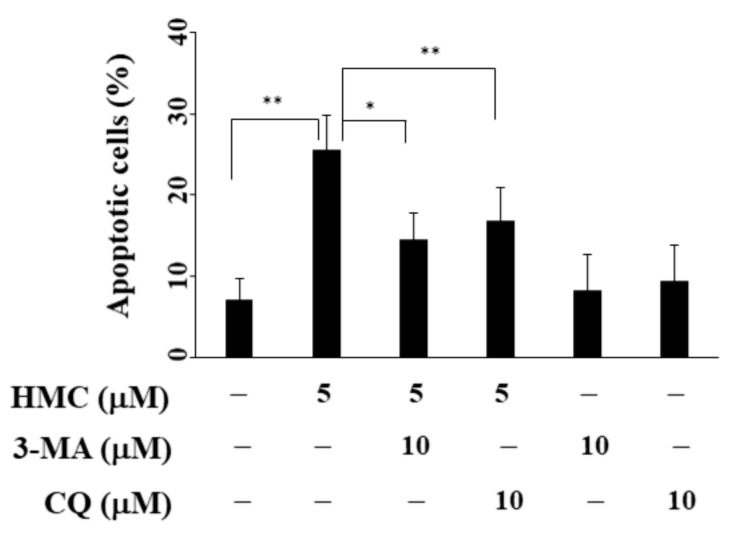
Co-treatment of autophagic inhibitor partially reversed HMC-induced apoptosis. MCF-7 cells were treated with HMC alone or in combination of 3-methyladenine (3-MA) or chloroquine (CQ) for 48 h and stained with propidium iodide (PI)/annexin V. SD (n = 4) * *p* < 0.05, ** *p* < 0.01.

To further investigate the role of autophagy in HMC-induced cell death, we examined the effect of pharmacological inhibition of autophagy on HMC-induced apoptosis in MCF-7 cells. As shown in Figure 6, co-treatment with the autophagic inhibitor 3-methyladenine A (3-MA) or chloroquine (CQ) could significantly reduce the extent of apoptosis induced by HMC.

## 3. Discussion

In the present study, we investigated the antitumor effect of a novel HDAC8-selective inhibitor HMC in breast cancer cells. In addition to inhibiting HDAC8 deacetylase activity (IC50 values of 200.7±0.3 nM and 798.4±0.3 nM using recombinant HDAC8 and HeLa nuclear extracts, respectively) [14], HMC could also downregulate HDAC8 expression in MCF-7 cells while not affecting HDAC1 expression. These data suggest that HMC might mediate its inhibitory effect on HDAC8 through two different mechanisms. Theses evidence suggests that HMC induced both apoptosis and autophagy in MCF-7 cells, and that concomitant treatment with autophagy inhibitors could attenuate HMC-induced apoptosis.

Although apoptosis is characteristic of pan-HDAC inhibitor-mediated anticancer effects [9,31,32], the role of HDAC8 in this programmed cell death event remains to be elucidated. In this study, we obtained evidence that selective inhibition of HDAC8 by HMC was effective in inducing mitochondria-dependent apoptosis, as manifested by Annexin V-PI staining, activation of caspase-3 and caspase-9, and PARP cleavage. Mechanistically, the proapoptotic effect of HMC shared many features of that of pan-HDAC inhibitors. For example, HMC was effective in inhibiting the Akt-mTOR signaling pathway, which led to increases in the expression levels of the proapoptotic protein Bax and decreased the expression of antiapoptotic proteins Mcl-1 and Bcl-2. Consistent with the reported role of pan-HDAC inhibitors in regulating the activity and expression of PPARγ [33,34], we also demonstrated the ability of HMC to enhance PPARγ transactivation activity and to modulate the expression of PPARγ and PPARγ-regulated gene products. These results suggested that Akt/mTOR and PPARγ signaling pathways might be partially responsible for the cell growth inhibition in HMC-treated MCF-7 cells.

ROS generation represents a major mechanism by which many therapeutic agents exert their antitumor effects [35,36]. Several reports showed that pan-HDAC inhibitors increased ROS levels in solid tumors and liquid tumors [23,37]. For example, Dahabieh et al. reported that SAHA induced apoptosis through increasing ROS generation in lymphoma cells [37]. Similarly, we also noted increased ROS-production in HMC-treated MCF-7 cells. As HDACs are known to potentiate DNA damage repair capacity, pan-HDAC inhibitors are potent inducers of DNA damage in transformed cells [38]. For example, the class I HDAC inhibitor depsipeptide caused DNA damage through ROS generation in cancer cells [39]. Our results demonstrated that HMC increased the phosphorylation of H2AX, an early response after the formation of DNA double strand breaks [35].

Autophagy, a cell recycling process, allows cells to survive from starvation and plays an important role in various physiological condition [40]. Dysregulation of autophagy led to diseases including neurodegeneration, aging, immunological diseases, and cancer [41,42]. Kundu et.al reported that targeting autophagy provides a viable strategy for the treatment of Alzheimer’s disease [42]. Due to the autophagy-inducing ability of HMC which suggested its potential as the treatment of inflammatory and neurodegenerative diseases which warrants further investigations.

It is found that knockdown of HDAC8 promotes autophagy which relates to the inhibition of growth in oral cancer cells [43]. We found that autophagy is an early response after the treatment of HMC for 6 h in MCF-7 cells. Previous studies have revealed that knockdown of Atg could increase the cytotoxicity of pan-HDAC inhibitors, which suggested that autophagy might serve as a prosurvival mechanism [44,45]. Our observation that autophagic inhibitors could protect cells from HMC-induced apoptosis is consistent with this notion [46,47]. Substantial evidence reveals that the potential mechanisms between autophagy and apoptosis including endoplasmic reticulum stress [48], PI3K/mTOR [49], and Bcl-2 [50] in cancer cells. A previous study showed that Bcl-2 would be displaced from Beclin-1 and Bax to induce autophagy and apoptosis under conditions of stress [51]. It’s possible that the ability of HMC to modulate the Akt/mTOR and Bcl-2 pathways plays a role in the crosstalk between autophagy and apoptosis.

In conclusion, our study showed that HMC induced caspase-dependent apoptosis via inhibition of Akt/mTOR signaling, caused DNA damage through ROS production, induced PPARγ activation and autophagy. Together, these findings suggest the potential of using HMC as a scaffold to develop potent HDAC8 inhibitors for breast cancer therapy.

## 4. Materials and Methods

### 4.1. Reagents, Chemicals, Antibodies

HMC was synthesized and characterized as previous report (S1, Figure S1) [14]. All agents were dissolved in DMSO, diluted in culture medium, and added to cells at a final DMSO concentration of 0.1%. The peroxisome proliferator-activated receptor response element (PPRE) x3-TK-Luc plasmids were purchased from Addgene (Cambridge, MA). Other chemicals and reagents were obtained from Sigma-Aldrich unless otherwise noted.

### 4.2. Cell Culture

Human breast cancer cell lines (MCF-7 and MDA-MB-231) were purchased from the American Type Culture Collection (Manasas, VA, USA). Non-tumorgenic human breast epithelial cell line (H184B5F5/M10) was kindly provided from Dr. Ming-Hong Tai (National Sun Yat-sen University). MCF-7 and MDA-MB-231 cells were cultured in DMEM/F12 (Invitrogen, Carlsbad, CA); and supplemented with 10% heat-inactivated fetal bovine serum (FBS; Gibco, Grand Island, NY) at 37 °C in a humidified incubator with 5% CO_2_. H184B5F5/M10 cells were maintained in α-MEM medium with the same supplements and culture condition.

### 4.3. Cell Viability Analysis

Cell viability of HMC was determined by 3-(4,5-dimethylthiazol-2-yl)-2,5-diphenyltetrazolium bromide (MTT) assays [20]. Briefly, 100 μL of 0.5 mg/mL MTT was added to each well plated 96-well plate and incubated for 4 h at 37 °C. Medium was removed and the reduced MTT dye was solubilized in 200 μL/well DMSO. A SPECTROstar Nano spectrophotometer (BMG LABTECH, Ortenberg, Germany) was used to measure the absorbance at 570 nm.

### 4.4. Flow Cytometry

For apoptosis assay, apoptotic cells were detected as described previously [52] using a commercial kit (BD Pharmingen, San Diego, USA) following the manufacturer’s instructions by flow cytometry (Attune NxT flow cytometer, ThermoFisher Scientific, Waltham, MA, USA). For caspase-3 activation, cells were seeded in 6-well culture plates and treated with DMSO or HMC at the indicated concentrations for 48 h. Then, the caspase-3 activity were assessed using a FITC rabbit anti-active caspase-3 kit (BD Pharmingen) according to the manufacturer’s protocol. ROS production were examined using the fluorescence probe 2’, 7’-dichlorodihyrofluorescein diacetate (H2DCFDA) [53].

### 4.5. Western Blot

Total cellular protein was isolated from the cells after various treatments. For Western blots, a previously described procedure was applied [54]. The following primary antibodies were used: Acetyl Histone H3, HDAC1, HDAC8, PPARγ, cyclin D1, CDK6, p-Ser^473^ Akt, Akt, p-Ser^2448^ mTOR, mTOR, p-Ser^139^ H2AX, H2AX, Bax, Mcl-1, PARP, procaspase-8, cleaved caspase-9, LC3B, and Atg5 were purchased from Cell Signaling Technologies (Beverly, MA, USA); β-actin, Sigma-Aldrich (St. Louis, MO, USA). The secondary antibodies were purchased from Santa Cruz Biotechnology. The enhanced chemiluminescence (ECL) system for detection of immunoblotted proteins was from GE Healthcare Bioscience (Piscataway, NJ, USA). Then, the protein was visualized by FUSION SOLO S (VILBER, Deutschland, Germany).

### 4.6. Acridine Orange Staining

MCF-7 cells (2 × 10^5^) were plated on coverslips and allowed to attach. Following treatment with DMSO (control) or HMC at the indicated concentration or rapamycin (100 nM) for 24 h, cells were stained with 1 μg/mL acridine orange for 15 min, washed with PBS, and examined under a ZEISS fluorescence microscope at ×200 objective lens magnification. The percentage of AVOs (dots with clear yellow or red fluorescence) was calculated using at least 100 cells per image in each condition under fluorescence microscopy.

### 4.7. Transient Transfection of PPARγ

Plasmids were transiently transfected into cells by using Fugene HD reagent (Roche, Mannheim, Germany) according to the manufacture’s protocol. After 24 h, transfected cell were treated with DMSO or HMC, and subjected to fluorescence analysis [20].

### 4.8. Statistical Analysis

All experiments were performed in three replicates. Statistical significance was determined with Student’s *t* test comparison between two groups of data sets. Differences between groups were considered significant at * *p* < 0.05, ** *p* < 0.01.

## Supplementary Materials

The following are available online at https://www.mdpi.com/2218-273X/9/12/824/s1, Figure S1: The synthetic procedure of HMC. Figure S2: Effects of 5 μM HMC or in combination of 5 mM *N*-acetylcysteine (NAC) or 500 μM glutathione (GSH) in MCF-7 cells for 24 h, and cell viability was determined by MTT assay.

## Abbreviations

histone deacetylases (HDACs); suberoylanilide hydroxamic acid (SAHA); trichostatin A (TSA); reactive oxygen species (ROS); peroxisome proliferator-activated receptor response element (PPRE); fetal bovine serum (FBS); phosphate-buffered saline (PBS); peroxisome proliferator-activated receptor (PPAR); autophagy-related (Atg); Yes-associated protein (YAP); acidic vesicular organelles (AVOs); autophagy-related (Atg); 3-methyladenine A (3-MA); chloroquine (CQ); *N*-acetylcysteine (NAC); glutathione (GSH).
