# Supplementary material for: Induction of Apoptosis and Autophagy in Breast Cancer Cells by a Novel HDAC8 Inhibitor
Source: Biomolecules. 2019 Dec 4;9(12):824. doi: 10.3390/biom9120824 (PMC6995545; doi:10.3390/biom9120824)

Supporting Information

Induction of apoptosis and autophagy in breast cancer cells by a novel HDAC8 inhibitor

Chang-Fang Chiu[1,2,3,†], Hsien-Kuo Chin[4,5,†], Wei-Jan Huang[6,†], Li-Yuan Bai[1,3], Hao-Yu Huang[5], and Jing-Ru Weng[5,6,7,8,*]

[1]Division of Hematology and Oncology, Department of Internal Medicine, China Medical University Hospital, Taichung 40447, Taiwan

[2]Cancer Center, China Medical University Hospital, Taichung 40415, Taiwan

[4]Division of Cardiovascular Surgery, Department of Surgery, Kaohsiung Armed Forces General Hospital, Kaohsiung 80284, Taiwan

[5]Department of Marine Biotechnology and Resources, National Sun Yat-sen University, Kaohsiung 80424, Taiwan

[6]Graduate Institute of Pharmacognosy, College of Pharmacy, Taipei Medical University, Taipei 11031, Taiwan

[3]College of Medicine, China Medical University, Taichung 40402, Taiwan

[7]Doctoral Degree Program in Marine Biotechnology, National Sun Yat-sen University, Kaohsiung 80424, Taiwan

[8]Graduate Institute of Natural Products, College of Pharmacy, Kaohsiung Medical University, Kaohsiung 80715, Taiwan

Supplementary Materials and Methods

S1: The synthetic procedure of HMC.

(*E*)-Ethyl 2-(3-chlorophenyl)-4-methoxycinnamate (2, the precusor of **HMC)**

To a mixture of **1** (300 mg, 0.85 mmol), 3,4-methylenedioxyphenylboronic acid (212 mg, 1.28 mmol) and K_2CO_3 (390 mg, 2.82 mmol) in anhydrous DMF (12 mL) was added $Pd(PPh_3)_4$ (163 mg, 0.141 mmol). The resulting solution was heated to 90 ^oC under N_2 for 3 h. After filtration with celite, the reaction mixture was concentrated in vacuo. The residue was diluted with EtOAc (50 mL) and then washed with distilled H_2O (40 mL x 3). The organic layer was dried (Na_2SO_4), filtered and the solvent was removed in vacuo. The residue was purified by column chromatography (silica gel; *n*-Hexane/EtOAc=5:1) to give **2** (200 mg, 72 %) as a solid. mp：85~87℃；^{1}H-NMR (300 MHz, $CDCl_3$) :δ= 7.69 (d, *J*=15.9 Hz, 1H), 7.63 (s, 1H), 6.93 (m, 2H), 6.86 (d, *J*=8.8 Hz, 1H), 6.84 (dd, *J*=8.8, 2.1 Hz, 1H), 6.80 (d, *J*=2.1 Hz, 1H), 6.29 (d, *J*=15.9 Hz, 1H), 6.03(s, 2H), 4.21 (q, *J*=7.1 Hz, 2H), 3.85 (s, 3H), 1.30 (t, *J*=7.8 Hz, 3H); ESI-MS m/z: 327 $[M+H]^+$.

(*E*)-*N*-Hydroxy-4-methoxy-2-(3,4-methylenedioxyphenyl)cinnamide (HMC)

NaOH (90 mg, 2.30 mmol) in 50% $NH_2OH_{(aq)}$ (2 mL) in an ice-bath was added to **2** (150 mg, 0.46 mmol) in THF/MeOH (3 mL: 3 mL). The residue was purified by column

chromatography (silica gel; CH_2Cl_2/ MeOH=33:1) to give **HMC** (100 mg, 65 %) as a solid. mp：138~141℃；^{1}H-NMR (300 MHz, [D_6]DMSO) : δ=10.63 (brs, 1H), 8.94 (brs, 1H), 7.61(d, *J*= 8.7 Hz, 1H), 7.31 (d, *J*= 15.6 Hz, 1H), 6.99 (m, 2H), 6.87 (d, *J*= 1.7 Hz, 1H), 6.86 (d, *J*= 2.7 Hz, 1H), 6.73 (dd, *J*=8.7, 1.7 Hz, 1H), 6.28 (d, *J*= 15.6 Hz, 1H), 6.09 (s, 2H), 3.81 (s, 3H);^{13}C -NMR (150 MHz, [D_6] DMSO) :δ=163.6, 160.2, 147.7, 147.4, 143.6, 137.0, 134.4, 128.2, 125.7, 123.6, 118.0, 115.5, 114.5, 110.3, 108.6, 101.7, 55.8 ppm; HRMS-ESI: m/z $[M-H]^-$ calcd. for $C_{17}H_{14}NO_5$: 312.0878, found: 312.0880.

S2: Effects of 5 μM HMC or in combination of 5 mM *N*-acetylcysteine (NAC) or 500 μM glutathione (GSH) in MCF-7 cells for 24 h, and cell viability was determined by MTT assay.

Cells (5x10^3) were treated with HMC (5 μM) in the presence of 5 mM NAC or 500 μM GSH in 5% FBS-supplemented DMEM/F12 medium in 96-well plates. After 24 h, the medium was removed, replaced by 200 µL DMEM/F12 containing 0.5 mg/mL of MTT, and cells were incubated in the CO_2 incubator at 37°C for 2 h. Supernatants were aspirated from the wells, and the reduced MTT dye was solubilized in 200 µL/well DMSO. Absorbance at 570 nm was measured using a plate reader.

Fig. S1. The synthetic procedure of HMC. Compound **1** was synthesized as previously described (Huang WJ et. al, ChemMedChem. 2012, 7, 1815-24). Suzuki coupling of compound **1** with boric acid in the presence of Pd(PPh3)4 gave **2**. Compound **2** reacted with NH_2OH produced HMC. a: $Pd(PPh_3)_4$, K_2CO_3, DMF, N_2, Δ; b: 50% $NH_2OH_{(aq)}$, NaOH, MeOH, THF, RT.

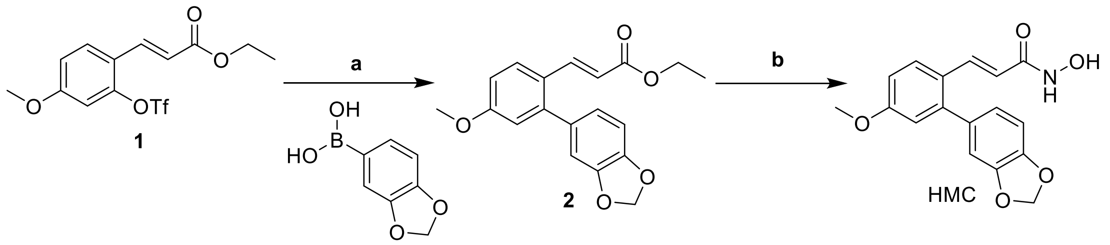

Fig. S2. Antiproliferative effects of HMC with or without *N*-acetylcysteine (NAC) or glutathione (GSH) in MCF-7 cells. Cell viability was determined by MTT assay at 24 h. *Points*, means; *bar*, S.D. (n = 4-6). ***P* < 0.01. NS, indicates not significant when comparing HMC alone treatment and the combined treatment of HMC and NAC or GSH.

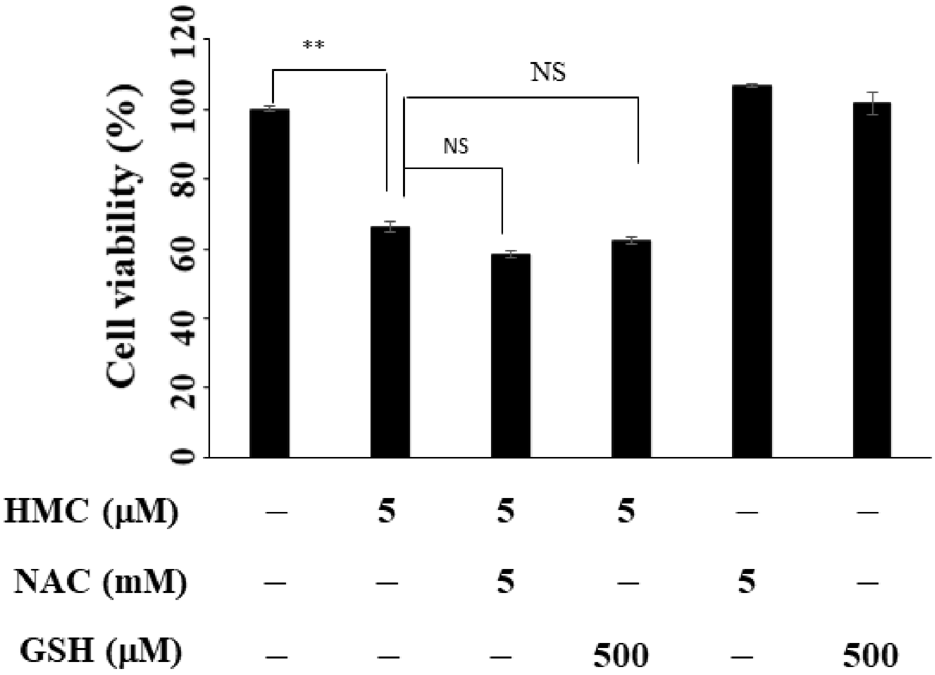

Supplement: Supplementary file 1 [file biomolecules-09-00824-s001.pdf]
